# Myo-inositol metabolism in two high-performance laying hen strains at different production periods

**DOI:** 10.1016/j.psj.2026.107571

**Published:** 2026-08-05

**Authors:** Ákos Szentgyörgyi, Vera Sommerfeld, Markus Rodehutscord, Korinna Huber

**Affiliations:** Institute of Animal Science, University of Hohenheim, Stuttgart, 70599, Germany

**Keywords:** *Myo*-inositol, Onset of egg laying, Insulin, Metabolic adaptation, Laying hen

## Abstract

This study aimed to investigate *myo*-inositol (**MI**) metabolism in two high-performance laying hen strains and to assess the effects of MI or mineral phosphorus supplementation at different production periods. Diets were corn-soybean meal-based and supplemented with MI (0, 1, 2, or 3 g/kg feed) in experiment 1 or with mineral phosphorus (0 or 1 g/kg feed) in experiment 2. Following a 4-week feeding period in metabolic units, ten hens per strain (Lohmann Brown-Classic and Lohmann LSL-Classic) and diet were euthanized at 30 weeks of age (experiment 1) and at 19 and 24 weeks of age (experiment 2). Plasma, liver, muscle, and kidney samples were collected from each hen. Tissue MI concentrations were measured using a commercial spectrophotometric kit, and concentrations of insulin and key enzymes (inositol monophosphatase and MI oxygenase) were quantified using ELISA kits. As diets did not affect tissue MI or enzyme concentrations, data from the two experiments were combined for further analysis. Changes across periods suggest that insulin influenced kidney MI oxygenase concentration and consequently played an essential role in the metabolic adaptation to egg laying. The results also indicated that higher liver and muscle MI concentrations in Lohmann Brown-Classic hens are potentially associated with metabolic adaptation to the onset of egg laying. Further research is needed to elucidate the role and regulation of MI in laying hens, particularly at the onset of egg laying.

## Introduction

*Myo*-inositol (**MI**) is one of nine possible stereoisomeric forms of the cyclic polyalcohol inositol. *Myo*-inositol can be obtained from multiple sources in different forms, including free MI, inositol-containing phospholipids, and phytate (Holub, 1986). In poultry nutrition, phytate occurs in plant feedstuffs, including corn and soybean meal (Kasim and Edwards, 1998). Phytate can be degraded by phytases and phosphatases, releasing phosphate and free MI, which are then absorbed in the gastrointestinal tract (Croze and Soulage, 2013; Holub, 1986; Sommerfeld et al., 2018). In a recent study with the same hens as in the present study, lower phytate concentrations were observed in the small intestine of laying hens when no mineral phosphorus was fed, suggesting enhanced phytate degradation (Sommerfeld et al., 2024). However, the primary source of MI is its endogenous synthesis from D-glucose, which, in rats, occurs mainly in the liver, brain, testis, and kidney (Eisenberg and Bolden, 1963; Hauser and Finelli, 1963). First, D-glucose is phosphorylated to D-glucose-6-phosphate by hexokinase, then converted to MI-1-phosphate by 1-D-MI-phosphate synthase, and finally, MI-1-phosphate is dephosphorylated to MI by the key enzyme inositol monophosphatase (**IMPase**) (Croze and Soulage, 2013). Catabolism of MI occurs mainly in the kidney (Howard and Anderson, 1967; Lewin et al., 1976), where MI is oxidized to D-glucuronic acid by MI oxygenase (**MIOX**). Through subsequent steps, D-glucuronic acid is converted to D-xylulose-5-phosphate, which can enter the pentose phosphate pathway to contribute to the production of NADPH and glycolytic intermediates (Croze and Soulage, 2013). Beyond its metabolism, MI plays an important role in cell signaling as a precursor of phosphatidylinositol, and is involved in energy and lipid metabolism, bone formation, skeletal muscle metabolism, and reproduction (Gonzalez-Uarquin et al., 2020c; Hammond and Burke, 2020).

*Myo*-inositol also exerts insulin-sensitizing effects, which have been observed in metabolic and endocrine disorders such as insulin resistance, diabetes mellitus, and polycystic ovarian syndrome in humans (DiNicolantonio and O'Keefe, 2022). In addition, MI functions as an organic osmolyte in mammalian kidneys (Sizeland et al., 1993). Despite extensive research on MI in mammalians, its physiological roles and effects on metabolism in chickens remain poorly understood.

Nevertheless, its effects on performance and metabolism in chickens have attracted increasing research interest (Pirgozliev et al., 2019; Gonzalez-Uarquin et al., 2020a; Cowieson and Zhai, 2021; Sprigg et al., 2022; Sommerfeld et al., 2025). In broiler chickens, studies on dietary MI supplementation have reported contradictory effects on growth performance (Sommerfeld et al., 2018; Pirgozliev et al., 2019; Cowieson and Zhai, 2021). In Lohmann Brown-Classic (**LB**) and Lohmann LSL-Classic (**LSL**) laying hens supplemented with 0, 1, 2 or 3 g MI/kg feed, no changes in body weight or average daily feed intake were observed (Sommerfeld et al., 2025). In addition to performance traits, studies have examined MI and key enzyme concentrations in broilers and laying hens. In Ross 308 broiler chickens, phytase supplementation at 6000 FTU/kg feed increased MI concentrations in the gizzard and ileal digesta, while no changes were observed in the liver or kidney (Sprigg et al., 2022). In another study with Ross 308 broiler chickens, phytase supplementation at 1500 and 3000 FTU/kg feed increased plasma MI concentrations; however, liver MI and IMPase, as well as kidney MIOX concentrations, did not differ among treatments, although kidney MI content was higher in the 1500 FTU/kg group compared to negative control (Gonzalez-Uarquin et al., 2020b). Further studies in Ross 308 broiler chickens have demonstrated that dietary phytase and MI supplementation alter tissue MI concentrations and influence the mRNA expression of genes involved in MI metabolism (Arthur, 2024). Supplementation with 30 g MI/kg feed increased MI content in muscle and kidney tissues. Similarly, supplementation with 4.5 and 13.5 g MI/kg feed increased MI concentrations in the kidney and jejunal digesta, while MI concentration in the jejunum was elevated following supplementation with 4.5 or 13.5 g MI/kg feed and phytase at 4500 FTU/kg feed. Furthermore, phytase supplementation at 4500 FTU/kg feed decreased kidney IMPase mRNA expression compared with the control and 4.5 g MI/kg feed groups, while supplementation with 4.5 g MI/kg feed increased kidney IMPase mRNA expression compared with the other treatment groups. In contrast, kidney MIOX, jejunal IMPase and MIOX mRNA expression remained unchanged following the different dietary supplementations (Arthur, 2024). A study with LB and LSL laying hens revealed differences in tissue MI, IMPase, and MIOX concentrations between different weeks of age during their productive lifespan and between laying hen strains (Gonzalez-Uarquin et al., 2021). Furthermore, correlations were observed between tissue enzyme levels and several plasma metabolites. These distinct findings suggest that the regulation of MI metabolism may depend on dietary supplementation, species and age. Therefore, the present study aimed to evaluate in two experiments the effects of dietary MI supplementation and dietary mineral phosphorus renunciation on MI metabolism in laying hens, and to investigate differences between LB and LSL hens and across three different production periods.

## Materials and methods

Experiment 1 (**Exp1**) was designed as a 2 × 4 factorial arrangement of treatments with two laying hen strains (LB and LSL) and four MI supplementation levels (0, 1, 2, or 3 g MI/kg feed) with *n* = 10 hens per group (in total 80 hens). Experiment 2 (**Exp2**) was designed as a 2 × 2 × 2 factorial arrangement of treatments with 2 laying hen strains (LB and LSL), 2 periods (19 and 24 weeks of age, representing before and after the onset of laying activity) and 2 levels of mineral phosphorus supplement (0 and 1 g phosphorus/kg) with *n* = 10 hens per group (in total 80 hens). Treatments were assigned in a randomized complete block design. The experiments took place at the Agricultural Experimental Station of the University of Hohenheim and was approved by the Regierungspräsidium Tübingen, Germany (Project no HOH67-21TE) in accordance with the German Animal Welfare Legislation.

### Animals and housing

The experimental procedures were described by Sommerfeld et al. (2024, 2025). Briefly, newly-hatched female chickens were obtained from a breeding company (Lohmann Breeders GmbH, Cuxhaven, Germany) and raised according to standard routine practices of the experimental station. In Exp1, at the age of 26 weeks, 10 hens (per diet and strain) were moved to metabolic units (size 1 m^3^; 1 hen per unit). In Exp2, at the age of 15 and 20 weeks (before and after the onset of egg-laying activity), 10 hens (per diet, strain, and period) were moved to metabolic units (size unit of 1 m^3^; 1 hen per unit). Metabolic units were equipped with a wire mesh floor, a nest box, a wooden perch, water cups, and a feeder. In Exp1, the photoperiod was 16 h of light and 8 h of darkness. In Exp2, the initial photoperiod was 9 h of light and 15 h of darkness until week 19, after which it was gradually adjusted to 16 h of light and 8 h of darkness by week 24. In both experiments, the temperature in the barn was set to 18 to 22°C.

### Diets

A detailed description of the diets was provided by Sommerfeld et al. (2024, 2025). In brief, the diets were based on corn and soybean meal to minimize intrinsic plant phytase activity. In Exp1, four diets were created based on the varying MI supplementation: without MI, or with 1 g/kg feed, 2 g MI/kg feed, or 3 g MI/kg feed. In Exp2, the hens used in the first period received a developer feed for the first 10 d after placement in the metabolic units at week 15, followed by a prelayer feed for 7 d, and a layer feed from d 17 until the end of the period. Each diet was provided either with or without mineral phosphorus supplementation. In the second period, hens received the same batch of layer feed from the day of placement in the metabolic units at week 20 until the end of the trial. The experimental diets were provided to hens individually housed in metabolism units for 4 weeks, starting at week 26 in Exp1, and at weeks 15 and 20 in Exp2. Feed and drinking water were provided for ad libitum consumption throughout the experiments.

### Plasma sampling

A detailed description of plasma sampling was provided by Sommerfeld et al. (2024, 2025). In Exp1, all birds were sampled at 30 weeks of age. In Exp2, 40 birds were sampled at 19 and 24 weeks of age, respectively. Hens were anesthetized with a gas mixture of 35% CO_2_, 35% N_2_ and 30% O_2_ and euthanized by bleeding. Blood samples from the trunk were collected into tubes containing EDTA (for metabolite profiling and insulin measures) or sodium fluoride (for MI determination). After collection, blood samples were centrifuged at 2,500 × *g* for 10 min at 4°C to obtain plasma. Thereafter, EDTA plasma was aliquoted and aliquots were shock-frozen in liquid nitrogen and kept on dry ice until stored at -80°C, while sodium fluoride plasma was stored directly at -20°C.

### Tissue sampling

After the blood sampling, hens were immediately eviscerated. Small samples of breast muscle, the right liver lobe (*Lobus hepatis dexter*), and the left kidney were collected using surgical equipment to preserve tissue integrity. The samples were washed in ice-cold physiological saline, cut into small pieces, shock frozen in liquid nitrogen, and collected in pre-chilled cryotubes. Tubes were kept on dry ice until storage at -80°C.

### Tissue homogenization

Breast muscle, liver, and kidney samples were ground in liquid nitrogen using a mortar and pestle. Powdered tissue (420 mg liver or kidney; 140 mg muscle) was mixed with 500 µl 1 × PBS containing protease inhibitor (cOmplete™, Mini; Roche, Basel, Switzerland) in lysing matrix tubes (SKU 115076200; MP Biomedicals, Irvine, CA, USA) with silica beads. Samples were homogenized in a FastPrep homogenizer (FastPrep-24™ 5G, MP Biomedicals, Irvine, CA, USA) at 6 m/s for 30 s, using 3 cycles for liver and kidney, and 4 for muscle. Homogenates were centrifuged at 1500 × *g* for 15 min (Eppendorf Centrifuge 5424 R; Eppendorf, Hamburg, Germany), and the supernatants were collected, aliquoted, and stored at -20°C for further analysis.

### Protein quantification

Liver, kidney, and breast muscle homogenates for other measurements were diluted at 1:500 in distilled water. Protein concentrations were determined by the method according to Bradford (Bradford Reagent, SERVA, Heidelberg, Germany) in triplicate for triglyceride (**TG**) determination and in duplicate for other measurements.

### *Myo*-inositol determination in liver, breast muscle, and kidney

*Myo*-inositol concentrations in different tissues were measured using a commercial spectrophotometric kit (K-INOSL; Megazyme, Bray, Ireland). Tissue homogenates were diluted in distilled water at ratios of 1:120 for liver, 1:50 for muscle, and 1:120 for kidney. Measurements were performed according to the manufacturer’s protocol, using an Infinite® 200 PRO microplate reader at 492 nm (Tecan Group Ltd., Männedorf, Switzerland). The assay was downscaled to 96-well microtiter plates (655101; Greiner Bio-One, Kremsmünster, Austria), with eight samples run per assay. All samples were assessed once, and concentrations were calculated using the standards provided by the kit. Final values were normalized to protein concentration (g/ml) to obtain units of milligrams MI per gram protein.

### Inositol monophosphatase 1 and *myo*-inositol oxygenase expression

Inositol monophosphatase 1 concentrations in the liver and muscle, and MIOX concentration in the kidney were measured using commercial ELISA kits (Chicken IMPA1 ELISA Kit, MBS7235623 and Chicken MIOX ELISA Kit, MBS7215577; MyBioSource, San Diego, CA, USA). Measurements were performed according to the manufacturer’s protocol using an Infinite® 200 PRO microplate reader (Tecan Group Ltd., Männedorf, Switzerland). A standard curve was constructed (concentrations on the x-axis and optical density on the y-axis) by using sigmoidal four parameter logistic (4PL) model in Magellan software (https://lifesciences.tecan.com/software-magellan), and IMPase and MIOX concentrations were calculated from this curve. All values were normalized to tissue homogenate protein concentration (mg protein/ml) to obtain units of picograms enzyme per milligram protein.

### Hexose determination in plasma

Plasma hexose concentrations were measured using the Absolute-IDQ^TM^ p180 kit (performed by Biocrates Life sciences AG, Innsbruck, Austria) and were re-used in this study as background data. The method and part of the results were reported by Szentgyörgyi et al. (2025), while additional results are based on unpublished data (Á. Szentgyörgyi, unpublished data).

### Insulin determination in plasma

The method and part of the results of plasma insulin determination were described previously (Szentgyörgyi et al., 2025), while additional results are based on unpublished data (Á. Szentgyörgyi, unpublished data). For the present study, the datasets from these experiments were reanalyzed and combined to provide a larger sample size. Plasma insulin concentrations were measured using a commercial ELISA kit (Chicken Insulin ELISA kit, MBS741090; Mybiosource, San Diego, CA, USA).

### Triglyceride determination in liver

The method and part of the results of liver TG were described previously (Szentgyörgyi et al., 2025), while additional results are based on unpublished data (Á. Szentgyörgyi, unpublished data). For the present study, the datasets from these experiments were reanalyzed and combined to provide a larger sample size. TG concentrations in the liver were measured using a commercial colorimetric kit (ab65336; Abcam, Cambridge, MA, USA). TG concentrations were given in nmol/mg protein.

### Bioinformatic and statistical analyses

The comparisons of treatments were performed using the MIXED procedure and pairwise t-tests using the software package SAS (version 9.4; SAS Institute Inc., Cary, NC). First, Exp1 and Exp2 were analyzed separately to assess the possible effects of the specific treatments. Since no significant diet effect was observed and hens were from the same strains in both experiments, the data from Exp1 and Exp2 were combined to examine the differences between three time points and between LB and LSL hens in a larger cohort. The individual hen was considered as the experimental unit. A detailed description of the models used for the separate analyses is provided in Sommerfeld et al. (2024, 2025). The following model was used for the combined analysis:

Y_ijkl_ = *µ* + *α*_i_ + *β*_j_ + (*αβ*)_ij_ + *γ*_k_ + *ϕ*_l_ + *ε*_ijkl_,

where Y_ijkl_ = response variable, µ = overall mean, α_i_ = effect of strain (fixed), β_j_ = effect of period (fixed), (αβ)_ij_ = the interaction between strain and period, γ_k_ = block (random), ϕ_l_ = father/rooster (random), and ε_ijkl_ = residual error. Pearson’s correlation analysis was performed to assess the linear associations between tissue MI, IMPase, and MIOX concentrations, as well as between plasma insulin and kidney MIOX concentrations using GraphPad Prism version 9.5.1 (GraphPad Software, La Jolla, CA, USA). Pathway analysis was performed using the Reactome Pathway Browser (https://reactome.org/PathwayBrowser/). *Myo*-inositol, IMPase, MIOX, glucose and insulin were used as input molecules to identify biological pathways associated with MI metabolism. The level of significance for all tests was set at *p* < 0.05. Values for the tables were given as LSmeans ± SEM.

## Results

Diets had no significant effect on IMPase, MIOX, and MI concentrations in either experiment; therefore, the datasets were combined, and diet was not considered in further analyses.

### Plasma hexose, insulin, and liver TG concentrations in the combined dataset from two experiments

Plasma hexose concentration decreased significantly over time (*P* < 0.001) in both strains (Table 1). Plasma insulin concentration reached a nadir at week 24 in both strains (week 24 vs. 19, *P* < 0.001; week 24 vs. 30, *P* < 0.001). In LB hens, the hexose:insulin ratio was not significantly different between weeks 19 and 24 (*P* = 0.111), but it was lower at week 30 than at week 24 (*P* = 0.002); whereas, in LSL hens, it peaked at week 24 (week 24 vs. 19, *P* < 0.001; week 24 vs. 30, *P* < 0.001). Liver TG concentration peaked at week 24 (week 24 vs. 19, *P* < 0.001; week 24 vs. 30, *P* < 0.001) and was higher at week 30 than at week 19 (*P* < 0.001) in both strains (Table 1).

**Table 1:** Plasma hexose and insulin concentrations, hexose:insulin ratio, and liver triglyceride (TG) concentration in two laying hen strains across three periods. Values are presented as LSmeans ± SEM (*n* = 160, except where noted). Different uppercase superscript letters (X,Y,Z) within a column indicate significant differences between periods *(P* < 0.01). Different lowercase superscript letters (a,b,c) within a column indicate significant interaction effects between period and strain *(P* < 0.05). Comparisons were made with the MIXED procedure using the software package SAS.

Strain	Period	Hexose (mmol/l)	Insulin^3^ (ng/ml)	Hexose:insulin ratio^4^	Liver TG^5^ (nmol/mg protein)
LB^1^	19 weeks	16.0	1.68	10.4^bc^	239
LB	24 weeks	14.0	1.18	13.6^b^	398
LB	30 weeks	12.3	1.84	8.16^c^	311
LSL^2^	19 weeks	16.4	1.84	9.73^bc^	216
LSL	24 weeks	13.7	0.94	18.8^a^	418
LSL	30 weeks	12.3	1.72	7.61^c^	333
pooled SEM		0.26	0.16	1.35	20.6
	19 weeks	16.2^X^	1.76^X^	10.1	227^Z^
	24 weeks	13.9^Y^	1.06^Y^	16.2	408^X^
	30 weeks	12.3^Z^	1.78^X^	7.89	322^Y^
LB		14.1	1.57	10.7	316
LSL		14.1	1.50	12.0	322
*P*-values					
Period		< 0.001	< 0.001	< 0.001	< 0.001
Strain		0.850	0.630	0.271	0.714
Interaction		0.410	0.501	0.049	0.490

1 LB = Lohmann Brown-Classic

2 LSL = Lohmann LSL-Classic

3 Insulin: *n* = 158

4 Hexose:insulin ratio: *n* = 158

5 Liver TG: *n* = 159

### Key enzyme and tissue MI concentrations in the two experiments separately

In Exp1, muscle IMPase (*P* = 0.046), kidney MIOX (*P* < 0.001), and muscle MI (*P* = 0.049) concentrations were higher in LB hens than in LSL hens (Supplementary Table 1). In Exp2, kidney MIOX concentration was significantly higher at week 19 than at week 24 (*P* < 0.001), while liver (*P* = 0.003), muscle (*P* = 0.036) and kidney MI (*P* = 0.006) concentrations were significantly lower at week 19 than at week 24. Liver IMPase and MI concentrations were significantly higher in LB hens than in LSL hens (*P* < 0.001) (Supplementary Table 2).

### Key enzyme and tissue MI concentrations in the combined dataset

None of the measured traits were affected by the interaction between period and strain. Liver IMPase concentration was higher at week 30 compared to week 19 and 24 in LB and LSL hens (week 30 vs. 19, *P* < 0.001; week 30 vs. 24, *P* = 0.006) (Table 2). LB hens expressed higher liver IMPase concentrations than LSL hens (*P* < 0.001). Muscle IMPase concentrations did not differ between weeks and strains. Kidney MIOX concentrations reached a nadir at week 24 (*P* < 0.001) with higher concentrations of MIOX in LB than in LSL hens (*P* = 0.016). A weak positive correlation was observed between plasma insulin and kidney MIOX concentration (Pearson’s r = 0.19, *P* = 0.043; *n* = 120 (3 statistical outliers were excluded from the analysis (data not shown)). Liver MI concentrations peaked at week 24 in LB and LSL hens (week 24 vs. 19, *P* = 0.010; week 24 vs. 30, *P* = 0.011) (Table 2), at a higher level in LB hens than in LSL hens (*P* = 0.013). Muscle MI concentrations expressed a peak concentration at week 24 in LB and LSL hens (week 24 vs. 19, *P* = 0.009; week 24 vs. 30, *P* = 0.001), at a higher level in LB hens than in LSL hens. Kidney MI concentrations also peaked at week 24 in LB and LSL hens (week 24 vs. 19, *P* = 0.003; week 24 vs. 30, *P* < 0.001), and strain differences were not detected.

**Table 2:** Inositol monophosphatase (IMPase), *myo*-inositol oxygenase (MIOX), and *myo*-inositol (MI) concentrations in the liver, muscle, and kidney of two laying hen strains across three periods. Values are presented as LSmeans ± SEM (*n* = 160, except where noted). Different lowercase and uppercase superscript letters (x,y,z) within a column indicate significant differences between periods at *P* < 0.05 and *P* < 0.01, respectively. Different lowercase and uppercase superscript letters (a,b) within a column indicate significant differences between strains at *P* < 0.05 and *P* < 0.01, respectively. Comparisons were made with the MIXED procedure using the software package SAS.

Strain	Period	Liver IMPase (pg/mg protein)	Muscle IMPase (pg/mg protein)	Kidney MIOX^3^ (pg/mg protein)	Liver MI (mg/g protein)	Muscle MI (mg/g protein)	Kidney MI^4^ (mg/g protein)
LB^1^	19 weeks	0.77	1.36	0.27	25.3	8.09	15.6
LB	24 weeks	0.85	1.43	0.07	29.1	12.8	18.6
LB	30 weeks	0.92	1.34	0.34	23.5	8.86	14.5
LSL^2^	19 weeks	0.67	1.22	0.20	19.8	8.27	13.6
LSL	24 weeks	0.69	1.39	0.03	23.9	8.93	17.8
LSL	30 weeks	0.84	1.09	0.15	22.7	7.12	14.3
pooled SEM		0.04	0.12	0.04	1.55	0.96	1.15
	19 weeks	0.72^Y^	1.29	0.24^X^	22.5^y^	8.18^Y^	14.6^Y^
	24 weeks	0.77^Y^	1.41	0.05^Y^	26.5^x^	10.9^X^	18.2^X^
	30 weeks	0.88^X^	1.21	0.25^X^	23.1^y^	7.99^Y^	14.4^Y^
LB		0.85^A^	1.38	0.23^a^	26.0^a^	9.91^a^	16.2
LSL		0.73^B^	1.23	0.13^b^	22.1^b^	8.11^b^	15.2
*P*-values							
Period		< 0.001	0.140	< 0.001	0.016	0.004	< 0.001
Strain		< 0.001	0.196	0.016	0.013	0.019	0.252
Interaction		0.613	0.543	0.071	0.115	0.143	0.673

1 LB = Lohmann Brown-Classic

2 LSL = Lohmann LSL-Classic

3 Kidney MIOX: *n* = 125

4 Kidney MI: *n* = 159

### Correlations between tissue key enzyme concentrations and MI concentrations in the combined dataset

Liver IMPase concentration was positively correlated with liver MI concentration (r = 0.48; *P* < 0.001). Similarly, muscle IMPase concentration was positively correlated with muscle MI concentration (r = 0.65; P < 0.001). No further significant correlations were observed (Supplementary Table 3).

### Pathway analysis

Pathway analysis identified an association of three input molecules (IMPase, MIOX, and MI) with the cytosolic synthesis of inositol diphosphate, inositol monophosphate, and inositol. The identified pathway had an entities *P*-value of < 0.001, and an entities FDR of < 0.001.

## Discussion

In this study, the term “period” refers to the time interval covering 1) ageing, growth, reproductive maturation, 2) the onset of egg laying, and 3) progression toward peak egg production. The diets did not have any effect on the measured traits. This was unexpected, as in the companion papers of Sommerfeld et al. (2024, 2025), an increased MI availability directly by MI feeding or indirectly, by reduction of mineral phosphorus content of the diet resulted in increased plasma MI concentrations. However, the initial hypothesis needs to be rejected; an increase in plasma MI did not affect the key enzymes of MI metabolism or tissue MI concentrations. Since the factor diet had no significant effect on the examined traits in both experiments, the discussion is based on the combined dataset comparing three ages and two strains. Furthermore, it is important to note that only tissue concentrations of IMPase and MIOX were measured in the present study, while their enzymatic activities were not measured. Therefore, the observed changes in tissue enzyme concentrations cannot be clearly interpreted as changes in enzyme activity, and the present findings should be considered with appropriate caution.

Changes in plasma hexose and insulin concentrations at different periods are most likely associated with the metabolic changes during the onset of egg laying. Decreased plasma hexose concentrations across periods may reflect increased utilization associated with enhanced egg-laying activity. Hexoses of the Biocrates metabolomics panel are mainly glucose. A physiological shift of glucose from plasma into the liver promoted TG synthesis, as reflected by the highest liver TG concentrations observed after the onset of egg laying. Liver lipid synthesis is the key process for egg yolk formation (Speake et al., 1998). The decrease in insulin and the increase in hexose:insulin ratio reflected that insulin secretion by pancreatic β-cells was reduced due to lower stimulation by blood glucose at the onset of egg laying, possibly as part of a physiological adaptation to egg production. Hexose:insulin ratio is analogous to the well-known fasting glucose:insulin ratio in humans, which is used as a measure of insulin sensitivity in women with polycystic ovarian syndrome (Legro et al., 1998); however, in our study, fasting values were not measured. Instead, conditions were standardized across both experiments, as feed was deprived for 1 h, followed by 1 h of ad libitum access to feed before slaughter. A similar adjustment of plasma glucose concentration has been reported in dairy cows after parturition (Bell and Bauman, 1997), suggesting a common principle of animals to start protein secretion. In hens, glucose is an important substrate for lipid synthesis and for energy production to synthesize egg yolk proteins during egg production (van Eck et al., 2023), while in cows, glucose is used by the mammary gland primarily for lactose synthesis.

A pattern similar to that of plasma insulin concentration was observed in kidney MIOX concentration. A significant positive correlation was found between these two traits, suggesting that plasma insulin may be associated with kidney MIOX concentration. In line with this assumption, insulin treatment in HK-2 cells (a renal proximal tubular cell line) resulted in increased MIOX expression in a dose-dependent manner (Tominaga et al., 2016). Thus, changes in insulin potentially resulted in changes in kidney MIOX concentrations, consequently in MI degradation, which may be essential for a successful metabolic adaptation to egg production. The knowledge about this association is scarce; however, since MI is an insulin-sensitizing agent in humans and other mammalians (Costantino et al., 2009; Croze et al., 2013; Antony et al., 2017), it could be hypothesized that decreased plasma insulin and kidney MIOX may have led to reduced MI degradation and increased MI accumulation, indicating a role of MI in the adaptation to the onset of egg laying.

Besides decreased catabolism, enhanced synthesis is another way to increase MI concentration in tissues. The concentration of MI-synthesizing key enzyme IMPase in the liver increased across periods. The knowledge about the regulation of IMPase, particularly in laying hens, is scarce. In a study using the HEK293T cell line, knockout of the IMPA1 gene, which encodes IMPase, resulted in a decreased recovery rate of phosphatidylinositol 4,5-bisphosphate levels at the plasma membrane, reduced carbachol-induced Ca^2+^ influx, and diminished intracellular Ca^2+^ release and subsequent store-operated Ca^2+^ entry compared with cells possessing an intact IMPA1 gene (Saha et al., 2023). Moreover, in IMPA1 knockout cells, lithium treatment did not affect the recovery rate of phosphatidylinositol 4,5-bisphosphate levels at the plasma membrane, carbachol-induced Ca^2+^ influx, or intracellular Ca^2+^ release, but it decreased the subsequent store-operated Ca^2+^ entry (Saha et al., 2023). Thus, it can be hypothesized that IMPase is essential for maintaining phosphatidylinositol 4,5-bisphosphate levels at the plasma membrane and for regulating intracellular Ca^2+^ influx and release. The increased liver IMPase concentration across periods may reflect enhanced regulation of intracellular Ca^2+^ or an elevated demand for MI associated with increasing egg production.

Changes in the tissue concentration of MI-degrading and MI-synthetizing enzymes were associated with changes in tissue MI concentrations, as supported by the observed positive correlations between IMPase and MI concentrations in the liver and muscle. A similar positive correlation was found between IMPase and MI concentrations in the liver in broilers receiving MI in ovo (Shomina et al., 2026). Furthermore, pathway analysis identified the involvement of IMPase, MIOX, and MI in cytosolic inositol phosphate metabolism, supporting the biological relationship among these molecules. Tissue MI concentrations were influenced by period with highest values at week 24, a period at which MIOX concentrations were the lowest. In animal models of diabetes mellitus, insulin resistance and hypertension, increased MIOX protein expression has been associated with elevated MIOX enzymatic activity (Chang et al., 2015). Similarly, in CD1 mice, a high-fat diet led to elevated MIOX protein expression and increased enzyme activity (Tominaga et al., 2016). If reduced MIOX concentration reflected decreased enzyme activity also in chicken and, consequently, reduced MI catabolism, the higher MI concentrations in tissues can be explained. LSL hens expressed lower MI concentrations in the liver and muscle than LB hens, suggesting a reduced requirement of MI in this strain. The present finding of lower kidney MIOX concentrations in LSL hens confirmed results from another study showing higher kidney MIOX concentrations in LB hens than LSL hens (Gonzalez-Uarquin et al., 2021). However, the time course of adaptation of MI metabolism is most likely associated with metabolic adaptation to egg production in both strains.

To further understand the physiological relevance of MI metabolism, it is important to consider the modulatory mechanisms involved in its regulation. Although MIOX activity was not measured in the present study, previous findings demonstrate that several factors may regulate MIOX activity, which should be considered when interpreting the present results. In LLC-PK1 cells, increasing D-glucose concentrations increased MIOX enzyme activity. Similarly, MIOX enzyme activity in the kidneys of 16-week-old diabetic *db/db* mice increased in proportion to serum glucose concentrations (Nayak et al., 2005). Furthermore, hyperglycemia increased both MIOX protein expression and enzymatic activity in the kidneys of streptozotocin-induced diabetic mice, and exposure of LLC-PK1 and HK-2 cells to increasing D-glucose concentrations resulted in dose-dependent increases in MIOX protein expression and activity. Moreover, kinase-mediated phosphorylation appears to regulate MIOX activity, as activation of protein kinase C, protein kinase A, and phosphoinositide-dependent protein kinase-1 increased MIOX phosphorylation and enzymatic activity, whereas kinase inhibition reduced these responses (Nayak et al., 2011). In addition, antioxidant treatment reduced the high glucose-induced increases in MIOX protein expression and enzymatic activity (Nayak et al., 2011). Furthermore, LLC-PK1 cells exposed to various hyperosmotic agents to induce hyperosmotic stress exhibited increased MIOX expression compared with the iso-osmotic controls (Prabhu et al., 2005). Overall, these findings may indicate that glucose availability, oxidative stress, and hyperosmotic stress may play an important role in the regulation of MIOX activity and MI metabolism. Notably, IMPase activity is mostly regulated by several positively charged ions, with Mg^2+^ being of the most important cofactors required for enzymatic activity (Hallcher and Sherman, 1980). In contrast, Ca^2+^ appears to be a competitive inhibitor, whereas Li^+^ inhibits IMPase activity through a noncompetitive mechanism (Hallcher and Sherman, 1980). Furthermore, the inhibitory effect of Li^+^ on IMPase might contribute to the induction of autophagy in various cells (Sarkar et al., 2005). These findings may indicate that IMPase activity is influenced by factors beyond enzyme abundance, which should be considered when interpreting changes in IMPase concentration. Overall, the regulation of MI metabolism and its interactions with other metabolic pathways are not well known, especially in chickens.

Previous findings demonstrated that dietary MI availability can change tissue MI concentrations, the concentrations of key enzymes involved in MI metabolism, and the mRNA expression of genes encoding these enzymes in broiler chickens and laying hens (Gonzalez-Uarquin et al., 2020b; Arthur, 2024). Changes in MI metabolism-related traits may be relevant for poultry production, as MI has insulin-sensitizing properties that may support metabolic health. Broiler breeders often exhibit disrupted ovarian follicle recruitment, a condition that has been suggested to resemble human polycystic ovarian syndrome (Walzem and Chen, 2014). In broiler breeder hens, treatment with metformin, an insulin-sensitizing agent similar to MI, appeared to support egg production (Weaver et al., 2025). Furthermore, supplementation with 3.8 g MI/kg feed increased plasma dopamine and serotonin concentrations in broilers, which may indicate reduced aggressive behaviour and feather pecking, thereby potentially improving animal welfare (Gonzalez-Uarquin et al., 2020; Huang et al., 2023). These potentially promising effects of MI require further investigation to determine whether MI supplementation can induce similar responses in poultry. Overall, a better understanding of MI metabolism could contribute to the development of precision feeding and management strategies to improve metabolic health, animal welfare, and optimize production performance.

To better understand the importance of MI metabolism, it is important to link the present findings to potential health and environmental challenges in poultry production. Reducing mineral phosphorus supplementation in laying hens may contribute to maintaining metabolic health while mitigating environmental impacts associated with excessive mineral phosphorus use. In the present study, dietary mineral phosphorus renunciation did not affect the measured components of MI metabolism or tissue MI concentrations. Therefore, dietary mineral phosphorus renunciation may represent a potential strategy to improve phosphorus utilization while maintaining MI homeostasis. A balanced MI metabolism may contribute to metabolic health, as MI acts as an osmolyte, serves as the structural basis for secondary messengers, and has insulin-mimetic properties (Croze and Soulage, 2013). Furthermore, maintaining MI homeostasis may influence the regulation of key enzymes in MI metabolism, including IMPase and MIOX. From a sustainability perspective, optimizing the release of MI and phosphorus from plant phytate may contribute to reducing mineral phosphorus supplementation and conserving non-renewable phosphorus resources. In a complementary study using the same animals as the present study, Sommerfeld et al. (2024) reported that phosphorus intake and retention were lower in the reduced dietary mineral phosphorus group compared with the supplemented group; however, phosphorus excretion did not differ between groups. Furthermore, performance traits were not affected by dietary mineral phosphorus renunciation. Therefore, reduced mineral phosphorus supplementation may be sufficient to maintain performance while improving phosphorus utilization in laying hens, at least during the examined period. This assumption is supported by Rodehutscord et al. (2023), who concluded that 2.2 g non-phytate phosphorus/kg feed is adequate to maintain laying performance. Similarly, in LB hens, reducing dietary mineral phosphorus from 3.8 to 2.2 g non-phytate phosphorus/kg feed did not affect laying performance or egg quality traits (Pagnussatt et al., 2025).

Environmental factors such as photoperiod and temperature could influence metabolic and hormonal processes and may also affect MI-metabolism. In the present study, photoperiod was 16 h of light and 8 h of darkness in Exp1 until week 30, while in Exp2 the initial photoperiod was 9 h of light and 15 h of darkness until week 19, after it was gradually adjusted to 16 h of light and 8 h of darkness by week 24. Furthermore, the temperature in the barn was set to 18 to 22°C in both trials. Thus, environmental conditions were comparable at week 24 and week 30, while a different photoperiod was applied at week 19. Although these differences do not suggest a direct association between photoperiod and the observed MI metabolism-related alterations, a potential modulatory effect of environmental conditions cannot be fully excluded. However, most of the differences in MI metabolism-related parameters were observed between the different periods, suggesting that physiological changes rather than photoperiod are the main drivers of the observed effects. Nonetheless, environmental changes should be considered when interpreting these findings.

To gain a more comprehensive understanding of the effects of MI supplementation, future studies should include long-term follow-up studies evaluating metabolic health, organ health, and production performance across different production stages. Although MI may represent a promising feed additive for poultry, its effects on performance have been inconsistent in broilers and laying hens. In a recent study, in ovo MI injection in broilers affected performance; however, MI metabolism-related traits remained unchanged (Shomina et al., 2026). These distinctions may be attributed to differences among experimental designs, including supplementation levels, animal age, and treatment duration. Therefore, future studies should focus on more uniform experimental designs, including longer supplementation periods (8-12 weeks) to provide more time for adaptation to experimental diets, and comparisons between low and high MI supplementation levels (e.g., 1 vs. 3 g MI/kg feed) in laying hens. Furthermore, evaluating organ health, particularly in the liver, gastrointestinal tract, kidney, reproductive organs, and brain, may provide further insight into the physiological effects and potential applications of MI supplementation. Furthermore, it would be valuable to evaluate these effects at the beginning of life, the onset of production, during peak egg laying, and at the end of the laying cycle to compare the effects of MI supplementation across different life stages characterized by different metabolic demands. To further improve the understanding and practical application of MI metabolism, modern technologies such as omics approaches, biotechnology, and genetic analyses may provide useful tools to further elucidate the regulation of MI metabolism.

In conclusion, plasma hexose, insulin concentrations, and hexose:insulin ratio suggested a reduced insulin secretion after the onset of egg laying, possibly as part of the physiological adaptation to the onset of egg production. In addition, the key enzyme in MI degradation, which is potentially strongly influenced by plasma insulin, appeared to have a central role in regulating MI concentrations in different tissues in LB and LSL hens. Finally, the underlying mechanisms and the role of insulin and MI in metabolic adaptation to egg production remain unclear, and further research is required to elucidate them.

## Author Contribution

Ákos Szentgyörgyi assisted with sample collection, performed the analysis and interpretation of the data, visualized the data and drafted the manuscript. Vera Sommerfeld assisted with sample collection, contributed to the design of the study, and performed the statistical modeling. Markus Rodehutscord and Korinna Huber contributed to the design of the study. Korinna Huber also contributed to the interpretation of the data. Vera Sommerfeld, Markus Rodehutscord, and Korinna Huber critically revised the manuscript for important intellectual content. All authors reviewed and approved the final version of the manuscript prior to submission for publication. All authors agree to be personally and publicly accountable for all aspects of the work.

## Disclosures

The authors declare the following financial interests/personal relationships which may be considered as potential competing interests:

Korinna Huber reports financial support was provided by German Research Foundation. If there are other authors, they declare that they have no known competing financial interests or personal relationships that could have appeared to influence the work reported in this paper.

## Footnotes

Supplementary material associated with this article can be found, in the online version, at doi:10.1016/j.psj.2026.107571.
