# Supplementary material for: Myo-inositol metabolism in two high-performance laying hen strains at different production periods
Source: Poult Sci. 2026 Aug 5;105(11):107571. doi: 10.1016/j.psj.2026.107571 (PMC13589128; doi:10.1016/j.psj.2026.107571)
Supplement: Supplementary file 1 [file mmc1.docx]

**Myo-inositol metabolism in two high-performance laying hen strains at different production periods**

Ákos Szentgyörgyi^1^, Vera Sommerfeld^1^, Markus Rodehutscord^1^ and Korinna Huber^1^

^1^Institute of Animal Science, University of Hohenheim, 70599 Stuttgart, Germany

**Supplementary table 1.** *Myo*-inositol (MI), inositol monophosphatase (IMPase), and MI oxygenase (MIOX) concentrations in the liver, muscle, and kidney of two laying hen strains fed either a basal diet or a basal diet with three levels of MI supplementation (MI0 = 0, MI1 = 1, MI2 = 2, MI3 = 3 g/kg feed). Values are presented as LSmeans ± SEM (*n* = 80, except where noted). Different lowercase and uppercase superscript letters (a,b) within a column indicate significant differences between strains at *P* < 0.05 and *P* < 0.01, respectively. Comparisons were performed with the MIXED procedure using the software package SAS

| Strain | Diet | Liver IMPase  (pg/mg protein) | Muscle IMPase  (pg/mg protein) | Kidney MIOX^3^  (pg/mg protein) | Liver MI  (mg/g protein) | Muscle MI  (mg/g protein) | Kidney MI  (mg/g protein) |
| --- | --- | --- | --- | --- | --- | --- | --- |
| LB^1^ | MI0 | 0.90 | 1.30 | 0.33 | 20.8 | 9.95 | 15.4 |
| LB | MI1 | 0.90 | 1.33 | 0.30 | 21.5 | 8.65 | 15.9 |
| LB | MI2 | 0.95 | 1.32 | 0.36 | 25.0 | 8.79 | 13.2 |
| LB | MI3 | 0.95 | 1.40 | 0.36 | 26.7 | 8.06 | 13.5 |
| LSL^2^ | MI0 | 0.79 | 1.23 | 0.08 | 19.6 | 6.81 | 13.4 |
| LSL | MI1 | 0.86 | 1.10 | 0.24 | 23.9 | 7.47 | 16.9 |
| LSL | MI2 | 0.76 | 0.91 | 0.15 | 21.7 | 6.36 | 13.6 |
| LSL | MI3 | 0.93 | 1.13 | 0.17 | 25.5 | 7.83 | 13.2 |
| pooled SEM |  | 0.07 | 0.20 | 0.05 | 2.53 | 1.12 | 1.49 |
| LB |  | 0.92 | 1.34^a^ | 0.34^A^ | 23.5 | 8.86^a^ | 14.5 |
| LSL |  | 0.84 | 1.09^b^ | 0.16^B^ | 22.7 | 7.12^b^ | 14.3 |
|  | MI0 | 0.84 | 1.26 | 0.21 | 20.2 | 8.38 | 14.4 |
|  | MI1 | 0.88 | 1.21 | 0.27 | 22.7 | 8.06 | 16.4 |
|  | MI2 | 0.86 | 1.12 | 0.25 | 23.3 | 7.58 | 13.4 |
|  | MI3 | 0.94 | 1.26 | 0.26 | 26.1 | 7.94 | 13.3 |
| *P*-values |  |  |  |  |  |  |  |
| Strain |  | 0.100 | 0.046 | 0.001 | 0.673 | 0.049 | 0.792 |
| Diet |  | 0.496 | 0.642 | 0.555 | 0.050 | 0.863 | 0.051 |
| Interaction |  | 0.593 | 0.639 | 0.325 | 0.566 | 0.427 | 0.638 |

^1^LB = Lohmann Brown-Classic

^2^LSL = Lohmann LSL-Classic

^3^Kidney MIOX: *n* = 70

**Supplementary table 2.** *Myo*-inositol (MI), inositol monophosphatase (IMPase), and MI oxygenase (MIOX) concentrations in the liver, muscle, and kidney of two laying hen strains at 19 and 24 weeks of age, fed diets without (P-) or with (P+) 1 g mineral phosphorus/kg feed supplementation. Values are presented as LSmeans ± SEM (*n* = 80, except where noted). Different uppercase superscript letters (X,Y) within a column indicate significant differences between periods *(P* < 0.01). Different uppercase superscript letters (A,B) within a column indicate significant differences between strains *(P* < 0.01). Comparisons were performed with the MIXED procedure using the software package SAS. No two- and three-way interactions were found, therefore the *p*-values were excluded from the table

| Strain | Period | Diet | Liver IMPase  (pg/mg protein) | Muscle IMPase  (pg/mg protein) | Kidney MIOX^3^  (pg/mg protein) | Liver MI  (mg/g protein) | Muscle MI  (mg/g protein) | Kidney MI^4^  (mg/g protein) |
| --- | --- | --- | --- | --- | --- | --- | --- | --- |
| LB^1^ | 19 weeks | P- | 0.73 | 1.30 | 0.33 | 24.0 | 7.50 | 18.1 |
| LB | 19 weeks | P+ | 0.82 | 1.42 | 0.22 | 26.5 | 8.67 | 13.0 |
| LB | 24 weeks | P- | 0.86 | 1.35 | 0.09 | 30.3 | 11.5 | 18.8 |
| LB | 24 weeks | P+ | 0.85 | 1.50 | 0.08 | 27.9 | 14.1 | 18.5 |
| LSL^2^ | 19 weeks | P- | 0.67 | 1.30 | 0.25 | 21.0 | 9.22 | 13.6 |
| LSL | 19 weeks | P+ | 0.67 | 1.13 | 0.14 | 18.5 | 7.31 | 13.5 |
| LSL | 24 weeks | P- | 0.68 | 1.34 | 0.03 | 24.7 | 8.04 | 19.8 |
| LSL | 24 weeks | P+ | 0.70 | 1.45 | 0.07 | 23.2 | 9.82 | 15.5 |
| pooled SEM |  |  | 0.05 | 0.15 | 0.04 | 1.88 | 1.77 | 1.85 |
| LB |  |  | 0.81^A^ | 1.39 | 0.18 | 27.2^A^ | 10.4 | 17.1 |
| LSL |  |  | 0.68^B^ | 1.30 | 0.12 | 21.9^B^ | 8.60 | 15.6 |
|  | 19 weeks |  | 0.72 | 1.29 | 0.23^X^ | 22.5^Y^ | 8.18^y^ | 14.6^Y^ |
|  | 24 weeks |  | 0.77 | 1.41 | 0.07^Y^ | 26.5^X^ | 10.9^x^ | 18.1^X^ |
|  |  | P- | 0.74 | 1.32 | 0.18 | 25.0 | 9.07 | 15.1 |
|  |  | P+ | 0.76 | 1.38 | 0.13 | 24.0 | 9.96 | 17.6 |
| *P*-values |  |  |  |  |  |  |  |  |
| Period |  |  | 0.176 | 0.254 | < 0.001 | 0.003 | 0.036 | 0.006 |
| Strain |  |  | < 0.001 | 0.434 | 0.158 | < 0.001 | 0.147 | 0.250 |
| Diet |  |  | 0.549 | 0.612 | 0.181 | 0.444 | 0.480 | 0.053 |

^1^LB = Lohmann Brown-Classic

^2^LSL = Lohmann LSL-Classic

^3^ Kidney MIOX: *n* = 55

^4^ Kidney MI: *n* = 79

**Supplementary table 3.** Pearson correlation coefficients among tissue concentrations of inositol monophosphatase (IMPase), *myo*-inositol oxygenase (MIOX), and *myo*-inositol (MI) in two laying hen strains at three periods with different dietary MI availability.

|  | Liver IMpase | Muscle IMPase | Kidney MIOX | Liver  MI | Muscle MI | Kidney MI |
| --- | --- | --- | --- | --- | --- | --- |
| Liver IMPase | 1 |  |  |  |  |  |
| Muscle IMPase | -0.15 | 1 |  |  |  |  |
| Kidney MIOX | 0.12 | -0.02 | 1 |  |  |  |
| Liver MI | 0.48^***^ | -0.03 | 0.08 | 1 |  |  |
| Muscle MI | 0.03 | 0.65^***^ | 0.02 | 0.02 | 1 |  |
| Kidney MI | -0.05 | 0.08 | 0.17 | 0.08 | 0.09 | 1 |

^***^*P* < 0.001
